# Right atrial and ventricular strain detects subclinical changes in right ventricular function in precapillary pulmonary hypertension

**DOI:** 10.1007/s10554-022-02555-6

**Published:** 2022-02-21

**Authors:** J. L. Vos, T. Leiner, A. P. J. van Dijk, H. B. van der Zwaan, G. Tj. Sieswerda, R. J. Snijder, M. C. Post, M. C. Vonk, S. van Leuven, P. Vart, M. Snoeren, A. Hirsch, S. El Messaoudi, R. Nijveldt, M. M. P. Driessen

**Affiliations:** 1 Department of Cardiology, Radboud University Medical Center, Geert Grooteplein 10, 6525 GA Nijmegen, The Netherlands; 2 Department of Radiology, University Medical Center Utrecht, Utrecht, The Netherlands; 3 Department of Cardiology, University Medical Center Utrecht, Utrecht, The Netherlands; 4 Department of Pulmonology, St. Antonius Hospital, Nieuwegein, The Netherlands; 5 Department of Cardiology, St. Antonius Hospital, Nieuwegein, The Netherlands; 6 Department of Rheumatology, Radboud University Medical Center, Nijmegen, The Netherlands; 7 Department of Health Evidence, Radboud University Medical Center, Nijmegen, The Netherlands; 8 Department of Radiology, Radboud University Medical Center, Nijmegen, The Netherlands; 9 Department of Cardiology and Radiology and Nuclear Medicine, Erasmus Medical Center, University Medical Center Rotterdam, Rotterdam, The Netherlands

**Keywords:** Pulmonary hypertension, Cardiovascular magnetic resonance imaging, Feature tracking, Right ventricular dysfunction

## Abstract

Right ventricular (RV) ejection fraction (EF) by cardiac magnetic resonance (CMR) correlates to outcome in precapillary pulmonary hypertension (pPH) patients, but is insensitive to early changes. Strain might provide incremental information. In this study, we compare right atrial (RA) and RV strain in pPH patients to healthy controls, and evaluate the prognostic value of strain in pPH. In this cross-sectional study, 45 pPH patients and 20 healthy controls underwent CMR, and feature-tracking derived RA and RV strain were evaluated. pPH patients had impaired RA reservoir and conduit strain, and RV longitudinal strain (LS), compared to healthy controls. In pPH patients with preserved RVEF (≥ 50%, n = 18), RA reservoir (35% ± 9 vs. 41% ± 6, p = 0.02) and conduit strain (16% ± 8 vs. 23% ± 5, p = 0.004), and RV–LS (−25% ± 4 vs. −31% ± 4, p < 0.001) remained impaired, compared to healthy controls. The association of strain with the primary endpoint (combination of all-cause death, lung transplantation, and heart failure hospitalization) was evaluated using a multivariable Cox regression model. RV–LS (HR 1.18, 95%–CI 1.04–1.34, p = 0.01) and RA strain (reservoir: HR 0.87, 95%–CI 0.80–0.94, p = 0.001; conduit: HR 0.85, 95%–CI 0.75–0.97, p = 0.02, booster: HR 0.81, 95%-CI 0.71–0.92, p = 0.001) were independent predictors of outcome, beyond clinical and imaging features. In conclusion, pPH patients have impaired RA strain and RV–LS, even when RVEF is preserved. In addition, RA strain and RV–LS were independent predictors of adverse prognosis. These results emphasize the incremental value of RA and RV strain analyses, to detect alterations in RV function, even before RVEF declines.

**Supplementary Information:**

The online version contains supplementary material available at 10.1007/s10554-022-02555-6.

## Introduction

Precapillary pulmonary hypertension (pPH) causes increased right ventricular (RV) afterload, inducing RV remodeling. The prognosis of patients with PH is not solely determined by pulmonary arterial pressure or pulmonary vascular resistance. Various studies have shown that the ability of the RV to adequately adapt to increased pressure loading is essential for a patient’s prognosis [1, 2]. If RV adaptation is not adequate, pPH will often result in RV dysfunction and dilatation, heart failure, and ultimately death. Right atrial (RA) dilatation and functional decline, caused by increased RV end-diastolic pressure and tricuspid regurgitation, is also associated with prognosis [1]. Cardiac magnetic resonance (CMR) imaging is an accurate and reproducible tool to assess RV morphology, volumes and ejection fraction (EF) and is considered the gold standard for noninvasive assessment of RV function [1, 3, 4]. However, RVEF is a global measure of RV function, and a decline in RVEF occurs at a late stage of pPH, when RV remodeling (hypertrophy and increasing contractility) falls short. Therefore, it is important to understand and recognize changes in RV function that occur before RVEF starts to decline [1]. CMR feature tracking (FT) is a promising post-processing technique that allows the assessment of changes in myocardial deformation on standard cine images [5], potentially useful to detect both global and segmental alterations in RA and RV function.

The aim of this study, therefore, is to evaluate RA and RV strain in relation to volume and function in pPH patients, in order to investigate whether alterations in myocardial deformation precede a decrease in RVEF, and to evaluate their prognostic importance.

## Methods

In this cross-sectional study, we included prevalent patients with pPH, consecutively enrolled in two different studies. The first study included pPH patients (n = 33) enrolled between August 2012 and November 2013 [6, 7]. The second study included patients with pulmonary arterial hypertension associated with systemic sclerosis enrolled between August 2019 and January 2020 (n = 12). In- and exclusion criteria were similar in both studies. The diagnosis of pPH was previously established on a cardiac catheterization, in accordance with the ESC/ESR guidelines [3]. Patients were recruited from three tertiary hospitals in the Netherlands. A group of healthy subjects of similar age and gender served as a control group (n = 20). The Ethical Review boards of all participating centers approved the study. Written informed consent was obtained from all study participants prior to inclusion. Demographic data and CMR were obtained in all subjects. Transthoracic echocardiography (TTE) and CMR were performed on the same day. TTE was only performed in the pPH patients. In addition, follow-up data was collected of pPH patients included in the first study (n = 33), making use of medical records. The primary endpoint was a composite endpoint of any major adverse cardiovascular event (MACE), defined as the combination of all-cause mortality, lung transplantation, and heart failure hospitalization. In case more than one endpoint in the same patient occurred, the most severe endpoint was used (death > lung transplantation > heart failure hospitalization).

### Echocardiography acquisition

The TTE was performed with a Toshiba Artida system (Toshiba) with a 5-MHz transducer, or a GE medical systems VIVID E9 (GE Healthcare) with a 1.5–4 MHz phased array transducer. Offline analysis was performed on commercially available software. RV systolic pressure (RVSP) was calculated by adding the Bernoulli equation derived pressure gradient from the maximum tricuspid regurgitation velocity to the estimated RA pressure [8]. RV fractional area change (FAC), defined as ([end-diastolic area − end-systolic area]/end-diastolic) × 100%, and tricuspid annular plane systolic excursion (TAPSE) by M-mode echocardiography, were measured on the apical 4-chamber view.

### CMR imaging acquisition

All study participants were scanned on commercially available 1.5 Tesla MR-scanners (Ingenia R4.1.2, Philips Healthcare; Siemens Avanto, Siemens Healthcare, GmbH; Signa or Discovery, GE medical systems). Standard cine images were acquired during repeated end-expiratory breath holds, using a balanced steady-state free precession sequence. Cine images were acquired with similar spatial resolution (typical voxel size 1.5 × 1.5 × 5 − 8 mm^3^, repetition time/echo time = 3.2/1.6, flip angle > 60°), and temporal resolution (30 phases per cardiac cycle, except for the long axis cine images in 3 study participants; in whom 25 phases per cardiac cycle, typically 25–30 ms). Consecutive short-axis cine images were acquired every 10 mm from base to apex up to measure RV (including the trabeculae) [6] and LV volumes and calculate EF.

### CMR feature tracking analysis

**Fig. 1:**
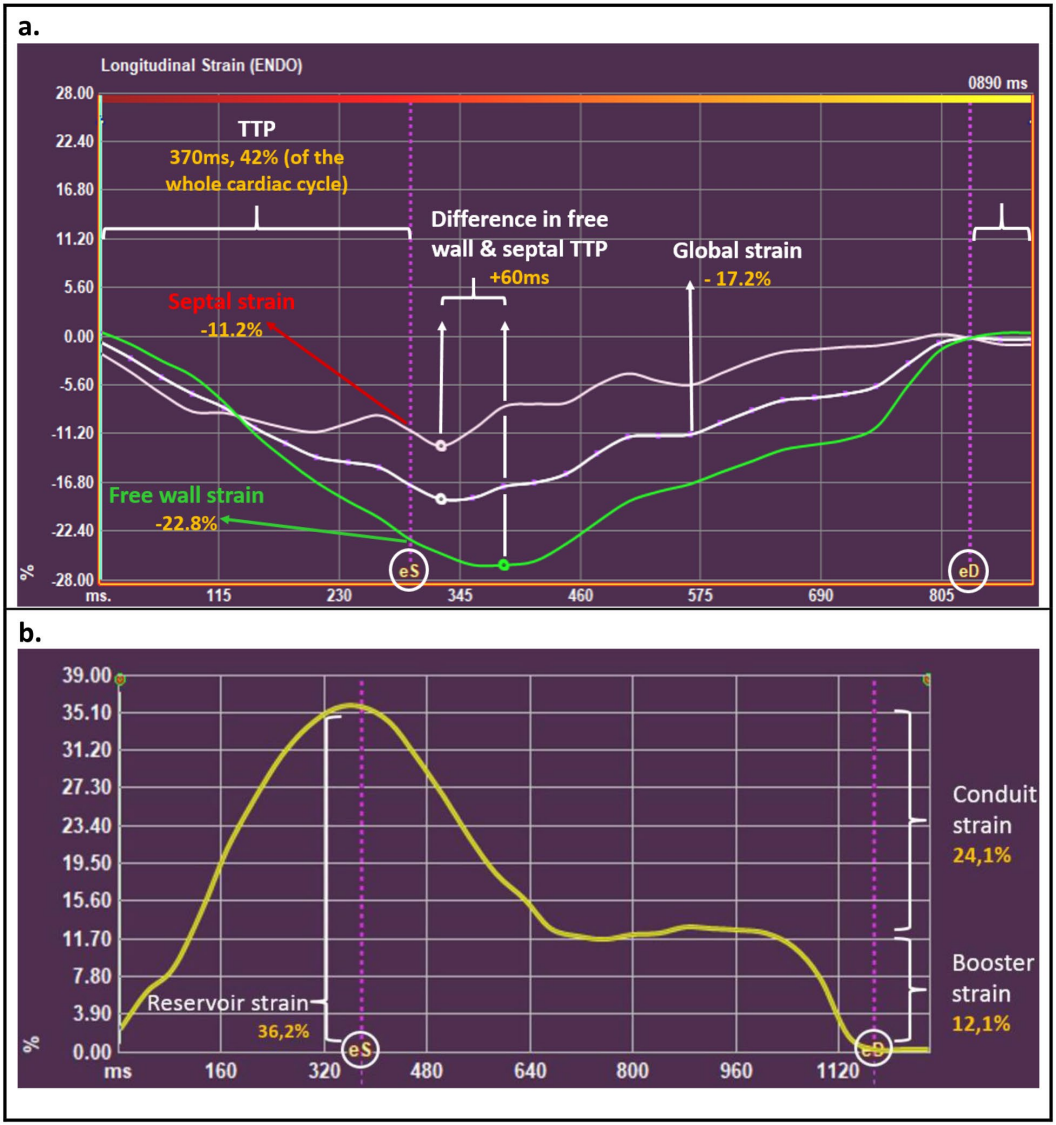
Depiction of the different strain parameters using Medis Qstrain software. **a*** Right ventricular longitudinal strain parameters.* Depiction of right ventricular (RV) longitudinal strain (%, y-axis) in time (ms, x-axis) in a precapillary pulmonary hypertension patient. Global, free wall and septal strain is measured at the time of eS (‘systolic strain’). eD is used as a reference to measure TTP. **b** *Right atrial strain parameters.* Depiction of right atrial (RA) strain (%, y-axis) in time (ms, x-axis). Reservoir strain measures the expansion of the RA (during RV systole) from the central venous return. Conduit strain is the passive filling of blood from the RA to the RV during early and mid-diastole. Booster strain measures the atrial contraction, in the late-diastolic, active, RV filling phase.  end-diastole;  end-systole; *TTP* time to peak strain

RV strain was measured using Medis Qstrain software (Medis Medical Imaging Systems, version 2.0.48.8.) on standard cine images. Endocardial contours were manually drawn in end-systolic and end-diastolic frame (defined as the smallest and largest RV volume, respectively), and automatically tracked in all other consecutive frames. Strain is computed by measuring the change in endocardial length between end-systolic and end-diastolic phase (as a percentage, with the end-diastolic length as reference) in a certain direction: Longitudinal strain (LS) in the longitudinal axis, circumferential strain (CS) when it is measured along the circumference. Global LS (GLS) was measured on the 4-chamber cines. CS was measured on the basal-, mid-, and apical ventricular short-axis cines, the average (global CS; GCS) is automatically calculated by Qstrain. RV contraction time was defined as time to peak strain (TTP). GCS was divided by GLS to assess GCS/GLS ratio. To detect mechanical discoordination, segmental LS (free wall-LS and septal-LS), and intraventricular delay (free wall TTP minus septal TTP) was analyzed. RA strain was measured on 4-chamber cines, and the reservoir (collecting the central venous return), conduit (passive filling of blood to the RV during early and mid-diastole) and booster strain (atrial contraction; active, late diastolic phase of the RV) was analyzed. RA volumes and EF, using the biplane Simpson’s area-length method, are automatically generated by Qstrain software [9]. RV LS and RA strain parameters are illustrated in Fig. 1. Strain analyses were performed by one single investigator (JV), supervised by a level III CMR-physician with > 15 years of experience (RN). To evaluate intraobserver (performed by JV, 2 weeks after the first analysis) and interobserver (performed by a second investigator, MD) variability, strain analyses were repeated in 30 CMR scans (10 scans of healthy controls, 20 scans of pPH patients).

### Statistical analyses

Continuous variables are presented as mean ± standard deviation (if normally distributed) or median (interquartile range) if not normally distributed. Categorical variables are presented as total number (percentage). Normality was assessed visually using Q–Q-plots and histograms. pPH patients and control group are compared using the independent sample t-test for normally distributed continuous data, or the Mann–Whitney U for not normally distributed continuous data. The Chi-Square test was used to compare categorical variables between groups. The intraclass correlation coefficient was used to measure inter- and intra-observer variability.

A sub analysis was performed to analyze whether FT can detect differences between healthy controls and pPH patients with a still preserved RVEF. A preserved RVEF is defined as RVEF ≥ 50% [10]. To analyze changes in RA and RV contraction in different stages of RV function, pPH patients were further divided into groups according to the RVEF (RVEF ≥ 50%, RVEF 40–50%, and RVEF < 40%) to get equally sized groups. A one-way analysis of variance (ANOVA) test or Kruskal Wallis test was used for comparison between healthy controls and the pPH groups stratified by RVEF. When ANOVA was significant, a post-hoc analysis was performed for pairwise comparison. Type I error rate was controlled using Dunnett’s test for multiple testing. Unadjusted and adjusted cox proportional hazards regression analyses were performed to evaluate the hazard ratio (HR, with 95% confidence interval; CI) of strain parameters. Strain parameters are included as continuous parameters. A selection of covariates that are previously suggested to be of clinical relevance (sex, age, WHO functional class, estimated RVSP, RVEF, and indexed RV end-diastolic volume [2, 11, 12]) were first univariably tested, and when significant added to the adjusted model. All statistical analyses were performed using SPSS (version 25). A P value less than 0.05 was considered statistically significant.

## Results

### Study population

In total, 20 healthy controls [aged 56 years (52–59), 20% male] and 45 patients with pPH [aged 58 years (46–72), 24% male] were included, characteristics shown in Table 1. In one pPH patient the CMR was not performed on the same day as TTE (15 days difference).

**Table 1:** Baseline characteristics of healthy controls and precapillary pulmonary hypertension patients

	Healthy controls (n = 20)	pPH patients (n = 45)	P value
Demographics			
Age (years)	56 [52–59]	58 [46–72]	0.34
Male, *n* (%)	4 (20%)	11 (24%)	0.76
Body surface area (m^2^)	1.8 ± 0.2	1.9 ± 0.3	0.09
Clinical classification of PH, *n* (%)			
Idiopathic PAH		15 (33%)	
PAH associated with systemic sclerosis		12 (27%)	
CTEPH		18 (40%)	
Duration of disease (years)		5.3 ± 5.4	
Treatment of PH, *n* (%)			
None		2 (4%)	
Monotherapy		15 (33%)	
Dual therapy		21 (47%)	
Triple therapy		7 (16%)	
Endotheline receptor antagonists		38 (64%)	
Prostanoids		9 (20%)	
Phosphodiesterase inhibitors		29 (64%)	
Riociguat		2 (4%)	
Oxygen therapy		9 (20%)	
Functional status assessment			
WHO Functional Class, *n* (%)			
I		3 (7%)	
II		29 (64%)	
III		12 (27%)	
IV		1 (1%)	
6 min walk distance (m)^a^		451 [391–528]	
NT-proBNP (pg/mL)		240 [111–1539]	

Values are in means ± SD, medians [interquartile range], or n (%)

*ACE* angiotensin-converting enzyme, *ARBs* angiotensin II receptor blockers, *CTEPH* chronic thromboembolic pulmonary hypertension, *ERAs* endothelin receptor antagonists, *PDIs* phosphodiesterase inhibitors, *PAH* pulmonary arterial hypertension, pPH precapillary pulmonary hypertension

^a^n = 43 (not available in 2 patients)

### Echocardiographic and CMR parameters

The mean estimated RVSP was 56 ± 19 mmHg in pPH patients (Table 2). To be noted, RVSP could not be estimated due to the lack of tricuspid regurgitation in 2 pPH patients. Due to poor image quality, FAC could not be measured in 11 patients (24%).

**Table 2:** Echocardiographic & CMR parameters of healthy controls & precapillary pulmonary hypertension patients

	Healthy controls (n = 20)	pPH patients (n = 45)	P value*	pPH patients with preserved RVEF (n = 18)	P value*
Echocardiography					
TAPSE (mm)		20 ± 4		21 ± 3	
RVSP (mmHg)^a^		56 ± 19		44 ± 11	
Fractional area change (%)^b^		31 ± 12		38 ± 11	
Cardiac magnetic resonance imaging					
LVEDV (mL)	136 ± 25	146 ± 35	0.28	151 ± 37	0.15
LVEDV-indexed (mL/m^2^)	77 ± 11	78 ± 16	0.79	80 ± 16	0.46
LVESV (mL)	55 ± 14	62 ± 23	0.25	57 ± 22	0.80
LVESV-indexed (mL/m^2^)	31 ± 6	33 ± 11	0.41	30 ± 11	0.74
LVEF (%)	60 ± 4	58 ± 9	0.40	63 ± 6	0.05
RVEDV (mL)	137 [117–163]	193 [172–273]	** < 0.001**	174 [133–203]	**0.03**
RVEDV-indexed (mL/m^2^)	81 [67–87]	101 [86–138]	** < 0.001**	94 [76–101]	**0.02**
RVESV (mL)	60 ± 20	140 ± 80	** < 0.001**	85 ± 33	**0.006**
RVESV-indexed (mL/m^2^)	33 ± 10	76 ± 50	** < 0.001**	44 ± 13	**0.006**
RVEF (%)	58 [54–62]	46 [38–53]	** < 0.001**	53 [50–57]	** < 0.001**
RV mass (g)	17 ± 4	36 ± 10	** < 0.001**	27 ± 10	** < 0.001**
RV mass-indexed (g/m^2^)	10 ± 2	19 ± 9	** < 0.001**	14 ± 4	** < 0.001**
RV fractional area change (%)	50 ± 5	36 ± 10	** < 0.001**	44 ± 7	** < 0.001**
RA maximum volume (mL)	85 [73–113]	127 [99–164]	** < 0.001**	99 [84–131]	0.08
RA minimum volume (mL)	47 [32–59]	71 [54–112]	** < 0.001**	54 [45–85]	**0.04**
RA volume prior to atrial contraction (mL)	71 [55–84]	105 [81–137]	** < 0.001**	82 [71–114]	**0.04**
RA EF (%)	49 ± 5	39 ± 12	** < 0.001**	44 ± 10	0.07

P-values in bold are statistically significant (p < 0.05)

Values are in means ± SD, medians [interquartile range], or n (%)

*EDV* end-diastolic volume, *EF* ejection fraction, *ESV* end-systolic volume, *LV* left ventricular, *pPH* precapillary pulmonary hypertension, *RA* right atrium, *RV* right ventricular, *RVSP* right ventricular systolic pressure, *TAPSE* tricuspid annular plane systolic excursion

*Compared to healthy controls

^a^Not available in 2 pPH patients

^b^Not available in 11 pPH patients

CMR derived LV volumes and EF were similar in pPH patients and healthy controls, whereas RVEF was impaired compared to healthy controls (Table 2). In addition, pPH patients had larger end-diastolic and end-systolic volumes, and higher RV mass. RA maximum and minimum volumes were higher in pPH patients (see Table 2 for values).

### RA and RV strain analysis

#### Reproducibility of RV strain parameters

The inter- and intra-oberserver variability of strain parameters (GLS and CS) were excellent, with intraclass correlation coefficients ranging from 0.84 to 0.94 (Table S1).

#### pPH patients versus healthy controls

**Fig. 2:**
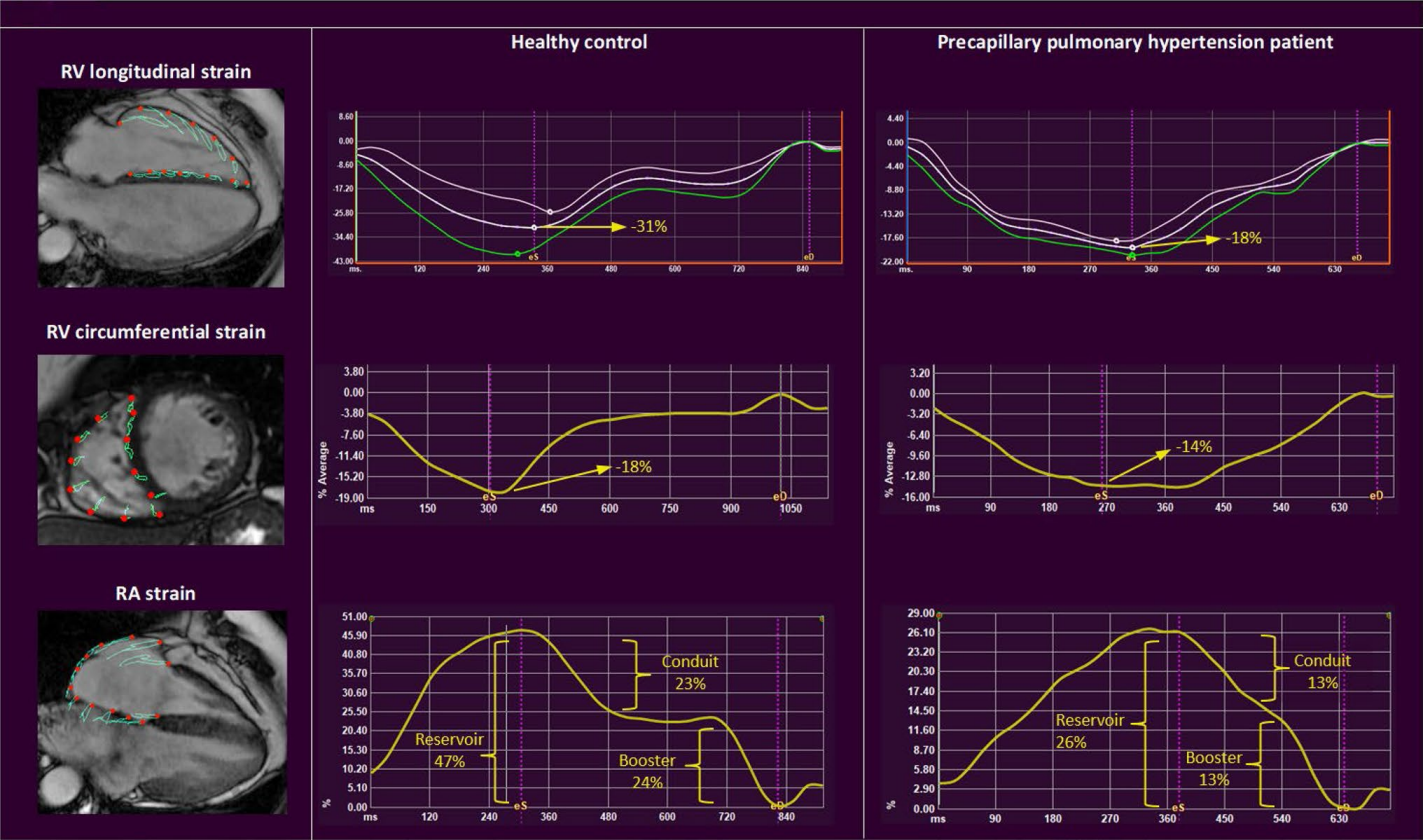
Representative images of right ventricular longitudinal and circumferential strain, and right atrial strain in a healthy control and a precapillary pulmonary hypertension patient. Representative images of the different strain parameters (%, y-axis) in time (ms, x-axis). Compared to the healthy controls, RV longitudinal (upper pictures) and circumferential strain (middle pictures) are lower in pPH patients. In addition, RA reservoir and conduit strain are lower in pPH patients compared to healthy controls, whereas there were no significant differences in RA booster strain (lower pictures). *pPH* pulmonary hypertension *RA* right atrial, *RV* right ventricular

Compared to healthy controls, pPH patients had lower RA reservoir and conduit strain, whereas RA booster strain was similar (see Table 3 for values). In one patient CS could not be measured (poor image quality). GLS and regional LS were significantly impaired in pPH patients compared to healthy controls. GCS, mid-CS and apical-CS were impaired in pPH patients compared to healthy controls, whereas basal-CS was similar in pPH patients and healthy controls. Compared to healthy controls, pPH patients had longer TTP contraction (% of the whole cardiac cycle). Figure 2 shows representative images of the RV and RA strain analysis in a healthy control and a pPH patient.

**Table 3:** CMR strain parameters of healthy controls & precapillary pulmonary hypertension patients

	Healthy controls (n = 20)	pPH patients (n = 45)	P value*	pPH patients with preserved RVEF (n = 18)	P value*
Right atrial strain					
Reservoir (%)	41 ± 6	29 ± 10	** < 0.001**	35 ± 9	**0.02**
Conduit (%)	23 ± 5	12 ± 7	** < 0.001**	16 ± 8	**0.004**
Booster (%)	18 ± 4	15 ± 7	0.10	19 ± 8	0.71
Right ventricular strain					
Global LS (%)	− 31 ± 4	− 20 ± 6	** < 0.001**	− 25 ± 4	** < 0.001**
TTP (as % of whole cycle)	40 ± 4	47 ± 7	** < 0.001**	48 ± 8	** < 0.001**
Free wall-LS (%)	− 39 ± 5	− 25 ± 8	** < 0.001**	− 31 ± 7	** < 0.001**
Septal-LS (%)	− 22 ± 5	− 14 ± 19	** < 0.001**	− 18 ± 4	**0.01**
Free wall & septum TTP difference (ms)	0 [0–0]	26 [− 27–59]	0.07	11 [− 27–29]	0.55
Global CS (%)^a^	− 15 ± 4	− 12 ± 5	**0.01**	− 15 ± 3	0.80
Basal CS (%)	− 12 ± 3	− 12 ± 4	0.68	− 15 ± 3	**0.001**
Mid CS (%)	− 14 ± 5	− 11 ± 5	**0.04**	− 13 ± 5	0.67
Apical CS (%)	− 21 ± 7	− 13 ± 9	**0.01**	− 17 ± 7	0.09
CS/LS ratio	0.49 ± 0.13	0.58 ± 0.24	0.12	0.62 ± 0.19	**0.02**

P-values in bold are statistically significant (p < 0.05)

Values are in means ± SD, medians [interquartile range], or n (%)

*CS* circumferential strain, *LS* longitudinal strain, *pPH* precapillary pulmonary hypertension, *RA* right atrium, *RV* right ventricular, *TTP* time to peak strain

*Compared to healthy controls

^a^pPH patients n = 44 (exclusion of one patient due to insufficient quality of the short-axis cines)

#### pPH patients with a preserved RVEF versus healthy controls

**Fig. 3:**
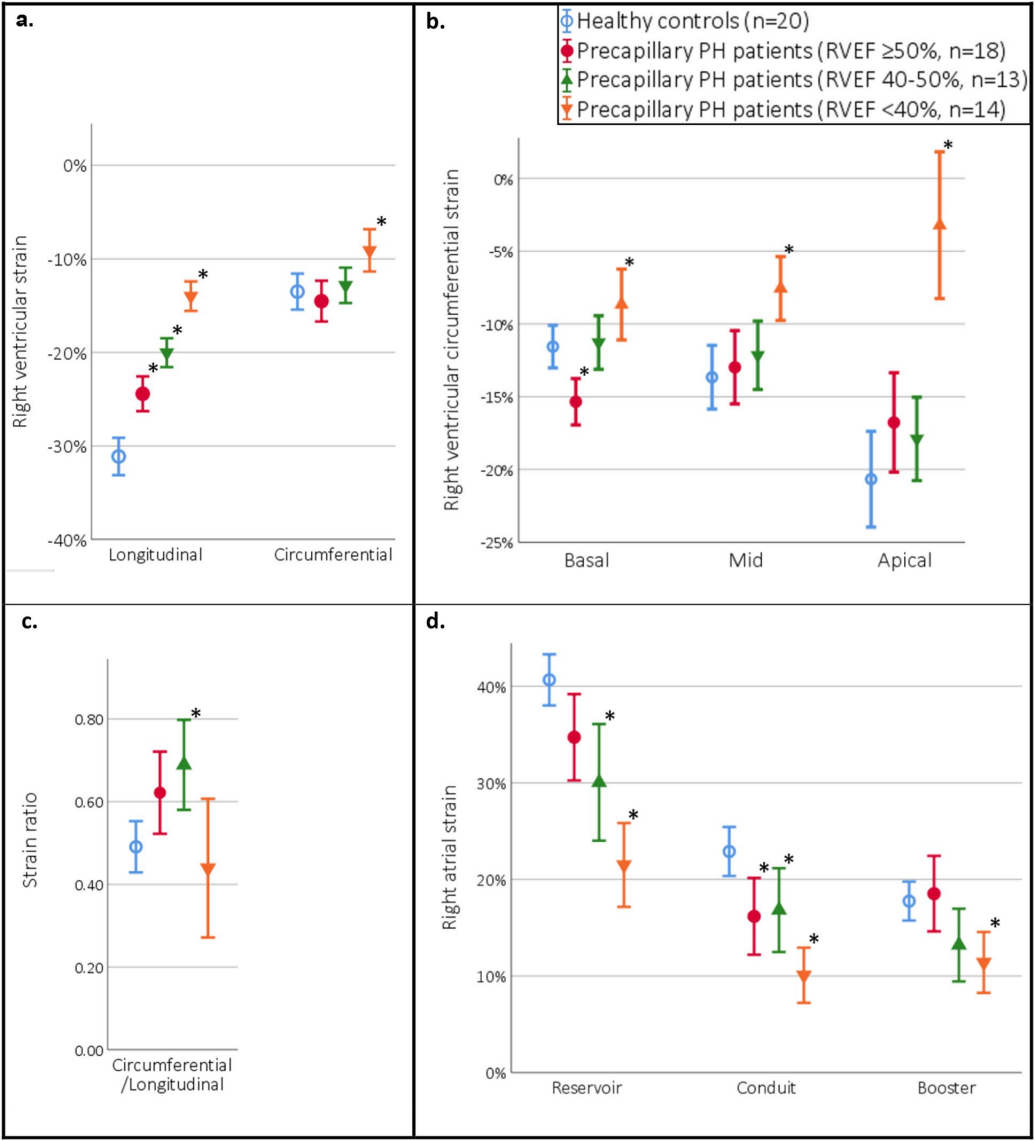
Right ventricular longitudinal and circumferential strain, and right atrial strain in healthy controls and precapillary pulmonary hypertension patients (stratified in groups by right ventricular ejection fraction). **a** *Right ventricular global longitudinal and global circumferential strain.* Compared to healthy controls, all pPH patient groups had lower global longitudinal strain, whereas circumferential strain was only lower in the pPH patient group with RVEF < 40%. **b** *Right ventricular basal, mid, and apical circumferential strain.* In pPH patients with preserved RVEF (≥ 50%) basal circumferential strain was better in comparison to healthy controls. In pPH patients with severely reduced RVEF (< 40%), circumferential strain was reduced on all levels. **c** Compared to healthy controls, patients with RVEF between 40 and 50% had a higher ratio between circumferential and longitudinal strain, in the other pPH patient groups no significant differences were found. **d** *Right atrial strain.* Compared to healthy controls, pPH patients had altered RA strain: all pPH patients had impaired conduit strain, and pPH patients with reduced RVEF (< 50%) had impaired reservoir strain. Booster strain was only significantly lower in pPH patient group with RVEF < 40%. *pPH* precapillary pulmonary hypertension, *RVEF* right ventricular ejection fraction. Bars are presented in means with error bars showing the 95% confidence intervals. *p < 0.05 compared to healthy controls

In total, 18 pPH patients had preserved RVEF (RVEF ≥ 50%). RA reservoir and RA conduit strain were lower in pPH patients, whereas booster strain was preserved (Table 3). GLS (− 25% ± 4 vs. − 31% ± 4, p < 0.001) was decreased, and TTP contraction longer in pPH patients with preserved RVEF compared to healthy controls. Compared to healthy controls, pPH patients still had a preserved GCS, consequently leading to a higher CS/LS ratio. Interestingly, pPH patients had significantly better basal-CS compared to healthy controls (− 15% ± 3 vs. − 12% ± 3, p = 0.001), and there was a trend toward worse apical-CS (− 17% ± 7 vs. − 21% ± 7, p = 0.09). In a multivariate analysis (including indexed-RV end-diastolic and end-systolic volumes, and RVEF), strain parameters were still independently associated with pPH (Table S2). The individual strain values of healthy controls and pPH patients are shown in Table S3. Figure 3 shows main RA and RV strain parameters in pPH patients stratified in groups by RVEF, compared to healthy controls.

#### The prognostic value of RA en RV strain to predict MACE in pPH patients

During a median follow-up of 8 [7–9] years, 12 (36%) out of the 33 pPH patients included in the first study reached the primary endpoint [all-cause death (n = 9), lung transplantation (n = 2), and heart failure hospitalization (n = 1)]. Only one patient, after > 3 years of follow up, was lost-to-follow up. All RV and RA strain parameters were associated with MACE, except for the RV CS parameters (for values see Table 4). After adjustment for the univariably significant covariates (WHO functional class ≥ 3 and indexed RV end-diastolic volume, see Table 4 for values), RV GLS and all RA strain parameters remained significant (Table 5).

**Table 4:** Univariable association with MACE

	pPH patients (n = 33)	
	Hazard ratio (95% CI)	P value
Age (years)	1.01 (0.97–1.05)	0.69
Sex (male)	1.02 (0.31–3.42)	0.96
WHO functional class 3 or 4	3.66 (1.15–11.71)	**0.03**
Estimated RVSP (mmHg)	1.01 (0.99–1.04)	0.33
RV end-diastolic volume-indexed (mL/m^2^)	1.01 (1.00–1.02)	**0.01**
RVEF (%)	0.96 (0.92–1.01)	0.14
RV global LS (%)^a^	1.18 (1.04–1.34)	**0.01**
RV global CS (%)^a^	1.01 (0.91–1.13)	0.80
RV CS Basal (%)^a^	1.06 (0.93–1.20)	0.39
RV CS Mid (%)^a^	1.02 (0.89–1.16)	0.82
RV CS Apical (%)^a^	1.00 (0.94–1.06)	1.00
Right atrial reservoir strain (%)	0.88 (0.83–0.95)	** < 0.001**
Right atrial booster strain (%)	0.86 (0.76–0.96)	**0.007**
Right atrial conduit strain (%)	0.85 (0.75–0.97)	**0.02**

P-values in bold are statistically significant (p < 0.05)

*CS* circumferential strain, *LS* longitudinal strain, *MACE* major adverse cardiovascular events, *RV* right ventricular, *RVEF* RV ejection fraction, *RVSP* RV systolic pressure

^a^Per + 1% strain increase (= less negative strain value, meaning strain becomes worse)

**Table 5:** Adjusted model for the prediction of MACE (adjusted for NYHA class ≥ III and indexed RV end-diastolic volume)

	All patients (n = 33)	
	HR (95% CI)	P value
RV global LS (per % increase)^a^	1.18 (1.04–1.34)	**0.01**
Right atrial reservoir strain (per % increase)	0.87 (0.80–0.94)	**0.001**
Right atrial booster strain (per % increase)	0.81 (0.71–0.92)	**0.001**
Right atrial conduit strain (per % increase)	0.85 (0.75–0.97)	**0.02**

P-values in bold are statistically significant (p < 0.05)

*LS* longitudinal strain, *MACE* major adverse cardiovascular events, *RV* right ventricular

^a^Per + 1% strain increase (= less negative strain value, meaning strain becomes worse)

## Discussion

In this study, we evaluated the alterations in RA and RV function using CMR-FT myocardial deformation in patients with pPH. In general, patients with pPH had lower RVEF, longer RV contraction times, impaired RA strain, RV GLS, and RV GCS compared to healthy controls. More importantly, we identified changes in RA and RV strain even in pPH with preserved RVEF. RA reservoir, and especially conduit strain, was lower in this group of pPH patients. RV GLS was impaired, while RV GCS was preserved. This resulted in a higher CS/LS ratio, meaning that RV function was more dependent on circumferential shortening in pPH (Central Illustration). RV GLS and all RA phasic strain parameters were independently associated with MACE, beyond clinical (age, sex, WHO functional class ≥ 3) and traditional imaging parameters (estimated RVSP, RVEF, and indexed-RV end-diastolic volume).

RV function is of utmost importance for a patient’s prognosis in pPH [1, 2]. CMR is the gold standard for noninvasive measurement of global RV function, measuring volumes and calculating RVEF [3, 13]. The recent development of CMR-FT makes it possible to measure RA and RV strain on standard cine images. In addition, inter- and intraobserver variability of CMR-FT is very low [14, 15], demonstrating good reproducibility. Echocardiography is widely available and inexpensive, and would therefore be the preferred method for measuring strain. However, a major drawback is the poor acoustic window, which limits RV strain imaging and lead to exclusion of data up to 12–17% [12, 16]. In our study, only one patient had to be excluded from CS measurement due to poor image quality.

The RV attempts to adapt to the increased pulmonary vascular resistance in pPH by increasing its contractility. RV remodeling, such as hypertrophy and changes in muscle properties, ensures that stroke volume can be maintained in the early stages. When these mechanisms fall short in later stages, the RV dilates and the heart rate increases, and RVEF decreases [1]. Since a decline in RVEF will only occur in later stages of heart failure, earlier markers of changed RV function would be valuable for guidance of medical therapy and follow-up of pPH patients. We believe that an impairment of RV FT-strain, recently shown to be of prognostic importance in other studies as well as ours [11, 17], might be such a marker. In addition, these parameters were able to identify changes in RV function, even in patients with preserved RVEF. In our study, RA conduit strain, reflecting passive filling of the RV, was lower in pPH patients with preserved RVEF compared to healthy controls. In a previous study by Tello et al. [18], RA strain was compared with invasive pressure–volume loop curves in PH patients, demonstrating that RA strain relates to RV end-diastolic pressure, reflecting diastolic function and stiffness of the RV, rather than RV contractility. This suggests that, similar to what is seen in the left ventricle [19], FT-strain is able to detect RV diastolic dysfunction.

RV GLS was also impaired in pPH patients with preserved RVEF, which is in line with previous studies, using fast strain-encoding imaging [20] as well as feature tracking derived strain [11, 17, 21]. CS is less frequently analyzed, but a recent study confirms our findings, demonstrating comparable GCS values in PH patients with preserved RVEF, and reduced GCS in PH patients with reduced RVEF [11]. This leads to higher CS/LS ratios in pPH patients in comparison to healthy controls. Under normal conditions, longitudinal shortening accounts for the majority of RV contraction [22]. It has been suggested that in pPH, RV function might be more dependent on circumferential shortening. RV remodeling induces a relative dominance of hypertrophic circumferentially oriented fibers, and RV dilatation creates a more transverse fiber orientation [11, 23, 24]. In addition, segmental CS analysis demonstrated interesting results, showing preserved basal-CS, and reduced mid- and apical-CS in pPH patients, the last being the most pronounced. This is similar to what is reported in a study of Kind et al. [23], measuring inward, radial movement, and a study of Fernandez-Friera et al. [25], measuring regional RVEF, both reporting apical function was most affected in PH, and even present in PH patients with preserved RVEF [25]. This early apical dysfunction could be attributed to the apex being especially thin-walled. Hence, apical dilatation, and thus reduced function, is the first to show up when RV pressure increases, and is even seen in acute RV afterload elevation, such as acute pulmonary embolism [26].

## Clinical implications

Although echocardiography is still the first line modality, CMR can provide incremental, relevant information in patients with proven PH, as it is the gold standard for noninvasive measurement of RV function [3]. CMR-FT analysis can be performed on standard cine images in a few minutes with excellent reproducibility. In total, it would take about 10–15 min of CMR-acquisition time and 10 min for post-processing analysis to obtain RA and RV volumes, calculate RVEF, and measure strain. These favorable characteristics make the implementation in clinical practice possible, e.g. for monitoring RA and RV function in follow-up of patients with PH. Future focus should focus on the clinical value of early RA and RV strain impairment—for guiding treatment strategy, prognosis, and possible improved screening for patients at risk of pPH.

## Limitations

This study has limitations. This multicenter study, performed at tertiary hospitals, has a limited sample size, which could make findings less generalizable to the general population. Although inter- and intra-observer variability was excellent in our study, it is known that CMR-FT intersoftware variability exists [27]. RA and RV GLS were of prognostic value, independent of all evaluated clinical covariates. However, caution must be applied when interpreting these results, since due to the limited sample size, not all possible confounders could be assessed, such as the heterogeneity in treatment during follow-up. In addition, since the CMR was performed at a single timepoint, we can only speculate about the changes in RV function that arise over time, and larger prospective longitudinal studies are needed to further evaluate these patterns, and to evaluate their prognostic significance.

## Conclusions

This study shows an impairment of RA and RV strain in pPH patients. More importantly, in pPH patients with a preserved RVEF, RA and RV strain parameters were able to identify changes in RV function, demonstrating altered RA strain and impaired RV GLS, whereas RV CS was preserved. This resulted in a higher CS/LS ratio, meaning that in pPH, RV function was more dependent on circumferential shortening. In addition, RV LS and RA strain are independent predictors of MACE, beyond clinical and imaging parameters. This study highlights the changes in contraction pattern that occur in pPH patients, even before global dysfunction is apparent. These results emphasize the incremental value of RA and RV strain analyses, to detect alterations in RV function even when RVEF is still preserved, and might be helpful to guide prognosis in pPH patients.

### Supplementary Information

Below is the link to the electronic supplementary material.

Supplementary file1 (DOCX 41 KB)

Supplementary file2 (PDF 477 KB)
