# Supplementary material for: Right atrial and ventricular strain detects subclinical changes in right ventricular function in precapillary pulmonary hypertension
Source: Int J Cardiovasc Imaging. 2022 Feb 21;38(8):1699–710. doi: 10.1007/s10554-022-02555-6 (PMC10509049; doi:10.1007/s10554-022-02555-6)
Supplement: Supplementary file 1 — Supplementary file1 (DOCX 41 KB) [file 10554_2022_2555_MOESM1_ESM.docx]

**Supporting information**

| **S1 Table**. Inter- and intraobserver variability of main strain parameters | | | | |
| --- | --- | --- | --- | --- |
|  | **Interobserver variability** | | **Intraobserver variability** | |
|  | ICC for absolute agreement | 95% CI | ICC for absolute agreement | 95% CI |
| RV longitudinal strain | 0.84 | 0.82-0.96 | 0.92 | 0.84-0.96 |
| RV circumferential strain | 0.91 | 0.81-0.96 | 0.93 | 0.86-0.97 |
| RV fractional area change | 0.92 | 0.86-0.97 | 0.86 | 0.84-0.97 |
| CI, confidence interval; ICC, intracorrelation coefficient; RV, right ventricular. | | | | |

| **S2 Table.** Strain parameters associated with precapillary pulmonary hypertension with preserved RVEF, adjusted for indexed RVEDV, indexed RVESV and RVEF | | | | |
| --- | --- | --- | --- | --- |
|  | Crude |  | Adjusted OR |  |
|  | OR (95% CI) | p-value | OR (95% CI) | p-value |
| *Right atrial strain parameters* |  |  |  |  |
| Reservoir (%) | 0.89 (0.80-0.99) | **0.03** | 0.91 (0.82-1.02) | ns |
| Conduit (%) | 0.87 (0.77-0.97) | **0.01** | 0.87 (0.77-0.98) | **0.03** |
|  |  |  |  |  |
| *Global RV strain parameters* |  |  |  |  |
| LS (%) * | 1.42 (1.13-1.78) | **0.002** | 1.42 (1.13-1.78) | **0.01** |
| TTP (as % of whole cycle) | 1.29 (1.08-1.53) | **0.004** | 1.28 (1.06-1.55) | **0.01** |
| LS/CS ratio | 1.06 (1.01-1.12) | **0.02** | 1.06 (1.01-1.13) | **0.03** |
| CS, circumferential strain; EDV, end-diastolic volume; EF, ejection fraction; LS, longitudinal strain; TTP, time to peak strain; RV, right ventricular.  * Per +1% strain increase (=less negative strain value, meaning strain becomes worse) | | | | |

| **S3 Table.** Individual values of the right ventricular ejection fraction and strain parameters of the study population | | | | | | | | | | |
| --- | --- | --- | --- | --- | --- | --- | --- | --- | --- | --- |
| Study ID | RVEF (%) | Global LS (%) | Global CS (%) | Basal CS (%) | Mid CS (%) | Apical CS (%) | CS/LS ratio (%) | RA reservoir strain (%) | RA conduit strain(%) | RA booster strain(%) |
| HC 1 | 56 | -26 | -13 | -11 | -10 | -18 | 0.5 | 30 | 16 | 14 |
| HC 2 | 63 | -35 | -14 | -8 | -15 | -20 | 0.4 | 38 | 24 | 14 |
| HC 3 | 62 | -35 | -16 | -10 | -13 | -26 | 0.5 | 40 | 26 | 14 |
| HC 4 | 62 | -33 | -22 | -16 | -20 | -31 | 0.7 | 33 | 14 | 19 |
| HC 5 | 55 | -29 | -13 | -7 | -18 | -14 | 0.4 | 40 | 28 | 11 |
| HC 6 | 61 | -31 | -16 | -12 | -10 | -26 | 0.5 | 41 | 20 | 20 |
| HC 7 | 67 | -31 | -18 | -12 | -19 | -24 | 0.6 | 48 | 24 | 24 |
| HC 8 | 54 | -31 | -19 | -12 | -9 | -35 | 0.6 | 49 | 28 | 21 |
| HC 9 | 66 | -32 | -19 | -16 | -16 | -26 | 0.6 | 50 | 25 | 24 |
| HC 10 | 57 | -33 | -13 | -13 | -13 | -13 | 0.4 | 34 | 17 | 17 |
| HC 11 | 59 | -28 | -17 | -11 | -17 | -23 | 0.6 | 34 | 17 | 16 |
| HC 12 | 54 | -25 | -18 | -9 | -19 | -26 | 0.7 | 44 | 24 | 20 |
| HC 13 | 55 | -28 | -15 | -16 | -13 | -17 | 0.5 | 46 | 28 | 18 |
| HC 14 | 58 | -34 | -15 | -12 | -12 | -23 | 0.4 | 45 | 20 | 24 |
| HC 15 | 56 | -39 | -7 | -10 | -4 | -10 | 0.2 | 44 | 34 | 10 |
| HC 16 | 54 | -27 | -10 | -9 | -9 | -14 | 0.4 | 41 | 29 | 12 |
| HC 17 | 62 | -41 | -18 | -16 | -22 | -17 | 0.4 | 46 | 24 | 22 |
| HC 18 | 51 | -28 | -20 | -15 | -17 | -28 | 0.7 | 36 | 15 | 21 |
| HC 19 | 69 | -31 | -13 | -13 | -14 | -14 | 0.4 | 38 | 21 | 17 |
| HC 20 | 52 | -25 | -7 | -5 | -7 | -11 | 0.3 | 39 | 23 | 17 |
| pPH patient 1 | 42 | -23 | -12 | -11 | -9 | -17 | 0.5 | 40 | 24 | 16 |
| pPH patient 2 | 37 | -16 | -7 | -12 | -5 | -4 | 0.4 | 31 | 22 | 9 |
| pPH patient 3 | 38 | -16 | -9 | -4 | -9 | -13 | 0.6 | 22 | 11 | 11 |
| pPH patient 4 | 20 | -12 | -2 | -1 | -7 | 4 | 0.1 | 14 | 7 | 8 |
| pPH patient 5 | 41 | -19 | -14 | -10 | -11 | -21 | 0.7 | 27 | 15 | 13 |
| pPH patient 6 | 50 | -21 | -13 | -17 | -12 | -10 | 0.6 | 36 | 10 | 26 |
| pPH patient 7 | 46 | -17 | -7 | -5 | -4 | -13 | 0.4 | 23 | 15 | 8 |
| pPH patient 8 | 45 | -25 | -14 | -12 | -14 | -17 | 0.6 | 41 | 16 | 25 |
| pPH patient 9 | 28 | -10 | -3 | -3 | -4 | -1 | 0.3 | 17 | 5 | 12 |
| pPH patient 10 | 52 | -22 | -13 | -13 | -12 | -16 | 0.6 | 28 | 15 | 13 |
| pPH patient 11 | 41 | -19 | -14 | -10 | -13 | -19 | 0.7 | 20 | 8 | 12 |
| pPH patient 12 | 38 | -15 | -13 | -13 | -13 | -15 | 0.9 | 13 | 7 | 5 |
| pPH patient 13 | 58 | -24 | -21 | -20 | -20 | -23 | 0.9 | 17 | 5 | 12 |
| pPH patient 14 | 47 | -19 | -17 | -15 | -13 | -21 | 0.9 | 25 | 11 | 14 |
| pPH patient 15 | 40 | -16 | -17 | -13 | -18 | -20 | 1.1 | 8 | 7 | 1 |
| pPH patient 16 | 47 | -21 | -14 | -15 | -13 | -14 | 0.6 | 39 | 33 | 6 |
| pPH patient 17 | 28 | -14 | -7 | -10 | -5 | -7 | 0.5 | 16 | 10 | 7 |
| pPH patient 18 | 19 | -11 | 1 | -5 | -3 | 10 | -0.1 | 27 | 7 | 19 |
| pPH patient 19 | 38 | -19 | -6 | -10 | -8 | -1 | 0.3 | 28 | 14 | 14 |
| pPH patient 20 | 37 | -17 | -2 | -11 | -4 | 8 | 0.1 | 32 | 18 | 14 |
| pPH patient 21 | 50 | -25 | -12 | -13 | -6 | -18 | 0.5 | 37 | 19 | 19 |
| pPH patient 22 | 32 | -12 | -8 | -11 | -11 | -2 | 0.7 | 15 | 7 | 8 |
| pPH patient 23 | 53 | -24 | -10 | -15 | -11 | -3 | 0.4 | 42 | 23 | 19 |
| pPH patient 24 | 50 | -22 | -18 | -11 | -16 | -26 | 0.8 | 22 | 11 | 11 |
| pPH patient 25 | 42 | -18 | -16 | -16 | -13 | -18 | 0.9 | 30 | 20 | 10 |
| pPH patient 26 | 23 | -12 | -5 | -11 | -8 | 5 | 0.4 | 32 | 9 | 23 |
| pPH patient 27 | 50 | -23 | -13 | -16 | -9 | -14 | 0.6 | 36 | 27 | 10 |
| pPH patient 28 | 44 | -21 | -11 | -13 | -8 | -14 | 0.5 | 24 | 10 | 13 |
| pPH patient 29 | 46 | -21 | -13 | -11 | -12 | -17 | 0.6 | 32 | 18 | 14 |
| pPH patient 30 | 23 | -11 | -4 | -6 | -6 | -1 | 0.4 | 25 | 8 | 17 |
| pPH patient 31 | 28 | -13 | -6 | -7 | -6 | -6 | 0.5 | 19 | 11 | 8 |
| pPH patient 32 | 72 | -29 | -19 | -18 | -19 | -22 | 0.6 | 34 | 19 | 15 |
| pPH patient 33 | 48 | -19 | -11 | -9 | -12 | -12 | 0.6 | 41 | 20 | 21 |
| pPH patient 34 | 56 | -22 | -13 | -15 | -10 | -15 | 0.6 | 36 | 19 | 17 |
| pPH patient 35 | 51 | -21 | -18 | -19 | -19 | -18 | 0.9 | 29 | 9 | 19 |
| pPH patient 36 | 56 | -26 | -10 | -8 | -13 | -11 | 0.4 | 30 | 22 | 9 |
| pPH patient 37 | 47 | -22 | -18 | -8 | -19 | -30 | 0.8 | 40 | 21 | 18 |
| pPH patient 38 | 30 | -18 | -18 | -16 | -17 | -22 | 1.0 | 11 | 5 | 6 |
| pPH patient 39 | 58 | -27 | -12 | -14 | -9 | -15 | 0.5 | 49 | 9 | 40 |
| pPH patient 40 | 56 | -30 | -16 | -19 | -16 | -15 | 0.5 | 38 | 9 | 29 |
| pPH patient 41 | 54 | -28 | -16 | -17 | -4 | -27 | 0.6 | 54 | 32 | 22 |
| pPH patient 42 | 53 | -30 | -13 | -16 | -13 | -8 | 0.4 | 42 | 30 | 12 |
| pPH patient 43 | 56 | -18 | -20 | -15 | -21 | -26 | 1.1 | 27 | 8 | 19 |
| pPH patient 44* | 61 | -35 | - | - | - | - | - | 36 | 12 | 23 |
| pPH patient 45 | 50 | -24 | -14 | -15 | -11 | -18 | 0.6 | 31 | 12 | 18 |
| CS, circumferential strain; EF, ejection fraction; HC, healthy control; LS, longitudinal strain; pPH, precapillary pulmonary hypertension; RA, right atrium; RV, right ventricular  * Shortaxis cine images of insufficient quality for CS analysis. | | | | | | | | | | |
