# Supplementary material for: Right atrial and ventricular strain detects subclinical changes in right ventricular function in precapillary pulmonary hypertension
Source: Int J Cardiovasc Imaging. 2022 Feb 21;38(8):1699–710. doi: 10.1007/s10554-022-02555-6 (PMC10509049; doi:10.1007/s10554-022-02555-6)
Supplement: Supplementary file 2 — Supplementary file2 (PDF 477 KB) [file 10554_2022_2555_MOESM2_ESM.pdf]

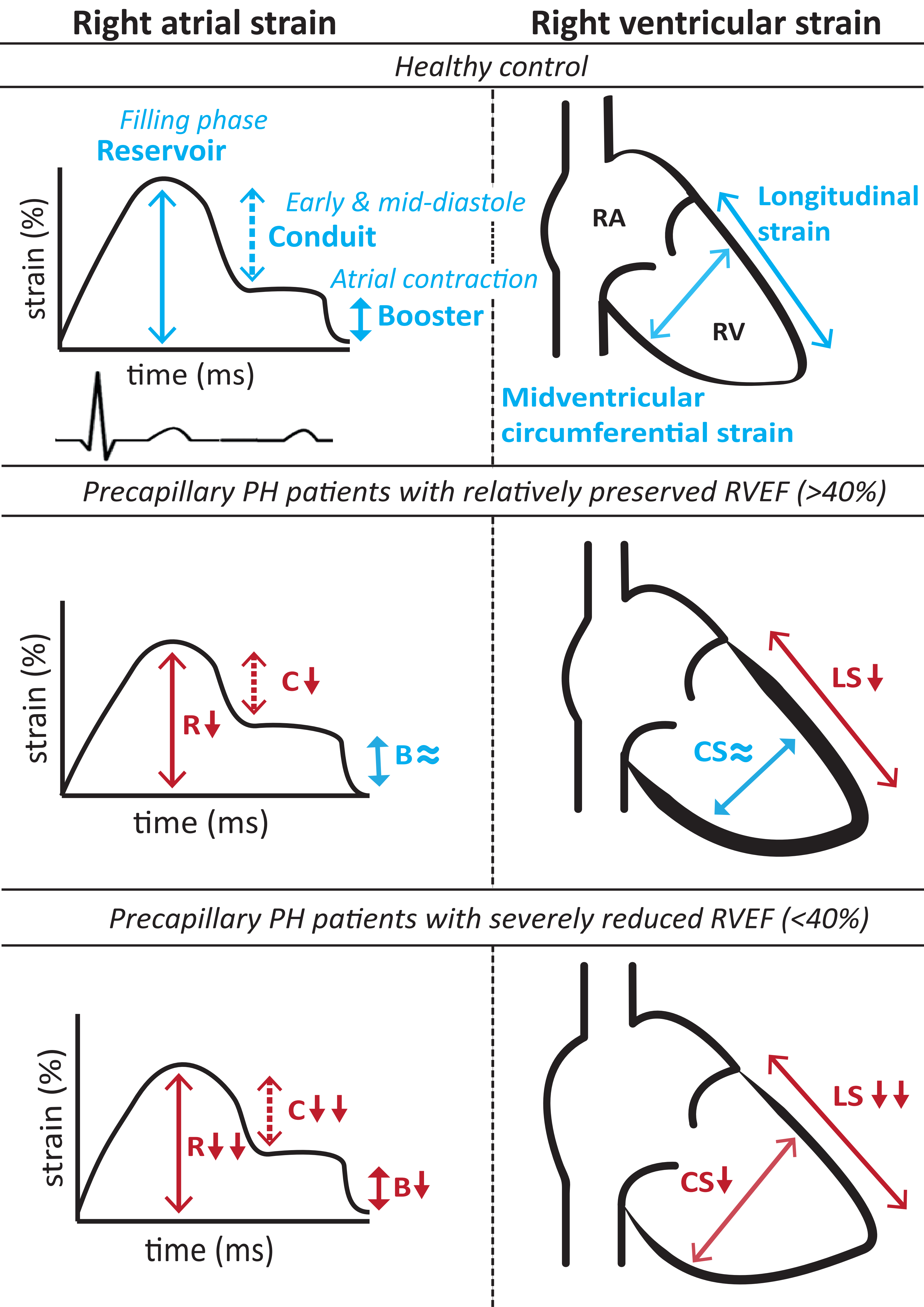

Right atrial strain
Right ventricular strain
Healthy control
Filling phase
Reservoir
Early & mid-diastole
Conduit
Atrial contraction
Booster
strain (%)
time (ms)
RA
RV
Longitudinal strain
Midventricular circumferential strain
Precapillary PH patients with relatively preserved RVEF (>40%)
C↓
R↓
B≈
LS↓
CS≈
strain (%)
time (ms)
Precapillary PH patients with severely reduced RVEF (<40%)
C↓↓
R↓↓
B↓
LS↓↓
CS↓
strain (%)
time (ms)
